# GDPD5 Related to Lipid Metabolism Is a Potential Prognostic Biomarker in Neuroblastoma

**DOI:** 10.3390/ijms232213740

**Published:** 2022-11-08

**Authors:** Tengling Luo, Junwei Peng, Qijun Li, Yao Zhang, Yun Huang, Lei Xu, Genling Yang, Dongmei Tan, Qian Zhang, Yi Tan

**Affiliations:** 1 Laboratory Animal Center, Chongqing Medical University, Chongqing 400016, China; 2 Ministry of Education Key Laboratory of Child Development and Disorders, Chongqing Key Laboratory of Pediatrics, Department of Pediatric Surgical Oncology, National Clinical Research Center for Child Health and Disorders, China International Science and Technology Cooperation Base of Child Development and Critical Disorders, Children’s Hospital of Chongqing Medical University, Chongqing 400016, China

**Keywords:** GDPD5, lipid metabolism, biomarker, neuroblastoma

## Abstract

Neuroblastoma (NB) is an extracranial solid tumor in children with poor prognosis in high-risk patients and its pathogenesis and prognostic markers urgently need to be explored. This study aimed to explore potential biomarkers related to NB from the aspect of lipid metabolism. Fifty-eight lipid metabolism-related differentially expressed genes between high-risk NB and non-high-risk NB in the GSE49710 dataset were analyzed using bioinformatics, including 45 down-regulated genes and 13 up-regulated genes. Gene Ontology (GO) and Kyoto Encyclopedia of Genes and Genomes (KEGG) analysis identified steroid hormone biosynthesis as an abnormal metabolic pathway in high-risk NB. Survival analysis established a three-gene prognostic model, including ACHE, GDPD5 and PIK3R1. In the test data, the AUCs of the established prognostic models used to predict patient survival at 1, 3 and 5 years were 0.84, 0.90 and 0.91, respectively. Finally, in the SH-SY5Y cell line, it was verified that overexpression of GDPD5 can inhibit cell proliferation and migration, as well as affect the lipid metabolism of SH-SY5Y, but not the sugar metabolism. hsa-miR-592 was predicted to be a potential target miRNA of GDPD5 by bioinformatics. In conclusion, this study develops a lipid-metabolism-related gene-based prognostic model for NB and demonstrates that GDPD5 inhibits SH-SY5Y proliferation and migration and may be targeted by hsa-miR-592 and inhibit SH-SY5Y fat synthesis.

## 1. Introduction

Neuroblastoma (NB) is a malignant childhood solid tumor derived from neuroectodermal cells of the sympathetic nervous system and is the leading cause of childhood cancer-related death [1]. Even when high-risk NB patients, especially those with metastases, are treated comprehensively, the prognosis remains poor. Thanks to various new patient stratification, new therapies and new drugs, the five-year survival rate for metastatic NB has improved dramatically over the past few decades [2]. Currently, the clinical outcome of NB is mainly predicted based on age, tumor stage, mitotic nuclear rupture index, MYCN (Neuroblastoma MYC Oncogene) amplification and ALK (Anaplastic Lymphoma Kinase) expression [3,4]. However, limitations remain in the risk stratification of NB patients. Therefore, there is an urgent need to explore new prognostic markers.

Recent studies have shown that lipid metabolism may be one of the important differences between cancer cells and normal cells and lipid metabolites may be important cancer markers. The growth, proliferation, invasion and metastasis of cancer cells can be affected by metabolic reprogramming, such as changes in lipid metabolism. This has been shown to occur in tumor cells and the tumor microenvironment [5,6,7]. Positron emission tomography in cancer patients has shown that NB tumors have high glucose uptake and high lactate production rates [8]. Moreover, inhibition of fatty acid synthesis results in increased neural differentiation and reduced tumor burden in NB xenograft experiments [9]. Rugolo et al. showed that very long-chain fatty acid protein 4 (ELOVL4) positively regulates neuronal differentiation and lipid droplet accumulation in NB cells and that high ELOVL4 expression is a marker of better overall clinical survival [10]. However, the roles and prognostic value of lipid-metabolism-related genes in NB remain to be elucidated, as the lack of a large-scale NB sample cohort limits the reliability and validity of previous findings. However, in the era of big data, the emergence of genome sequencing technology and data may help tumor diagnosis and prognosis prediction [11,12].

GDPD5 is a glycerophosphocholine phosphodiesterase identified in a family of bacterial genes [13], which is expressed in neurons, terminally differentiated oligodendrocyte subsets and vascular endothelium [14,15,16] and is critical for neuronal differentiation [17], growth and survival [18]. Recent studies have shown that GDPD5 promotes NB differentiation by releasing glypican [19].

In this study, the expression profiles of lipid-metabolism-related genes in the Gene Expression Omnibus (GEO) database (GSE49710 dataset) were downloaded and analyzed to identify differentially expressed genes in high-risk versus non-high-risk NBs. Gene Ontology (GO) and Kyoto Encyclopedia of Genes and Genomes (KEGG) analysis showed that genes were mainly involved in steroid hormone biosynthesis. Next, using the Least Absolute Shrinkage and Selection Operator (LASSO) and the multivariate Cox algorithm, we developed a risk score for predicting the prognosis of NB patients using the GSE49710 dataset. Finally, we validated the correlation of GDPD5 in risk scoring models with lipid metabolism in tumor cells and tumor malignancy through biological experiments. In conclusion, we built a three-gene trait model that can be used as a predictor of NB survival. In addition, the effects of GDPD5 on lipid metabolism, proliferation and migration of NB were verified by biological experiments.

## 2. Results

### 2.1. Identification of Differentially Expressed Genes Related to Lipid Metabolism between High-Risk Groups and Non-High-Risk Groups and Patient Survival in Neuroblastoma (NB)

**Figure 1:**
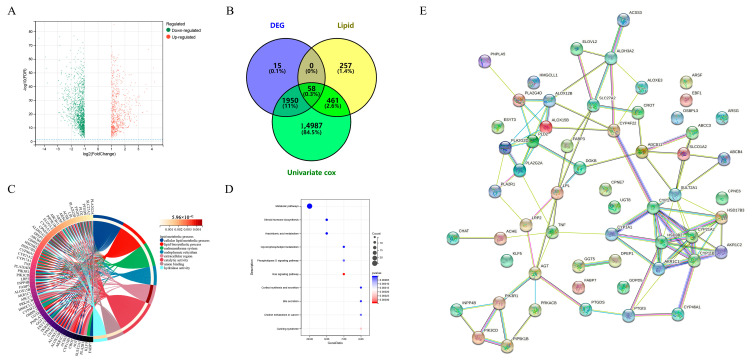
Discovery of differentially expressed genes and aberrant metabolic pathways in high-risk neuroblastoma. (**A**) Volcano plots of differentially expressed genes (DEGs) in the GSE49710 dataset. The x-axis represents fold change in gene expression and the y-axis represents FDR. The red and green dots in the graph represent statistically significant up- and down-regulated genes. (**B**) Venn diagrams of DEG, lipid-metabolism-related genes and genes screened by univariate CCox. (**C**) Gene ontology (GO) analysis of 58 genes. (**D**) Kyoto Encyclopedia of Genes and Genomes (KEGG) analysis of 58 genes. (**E**) Protein–protein interaction (PPI) network of 58 genes.

According to the method of Zheng et al., human lipid-metabolism-related pathways were downloaded from the molecular signature database (version 7.0). Herein, a total of 776 lipid metabolism genes was sorted out from the six lipid metabolism pathways from the databases KEGG and Reactome [20]. We obtained a total of 776 lipid metabolism genes (Table S1). We then explored differentially expressed genes between high-risk and non-high-risk NB samples in dataset GSE49710. The cohort and clinicopathological information in the 498 NB samples of the GSE49710 dataset are shown in Table 1. We found 58 lipid-metabolism-related genes that were significantly associated with NB risk and survival (Figure 1A,B), of which 13 were up-regulated and 45 were down-regulated. GO analysis showed that these genes were mainly involved in a cellular lipid metabolic process and lipid metabolic process, most of which were located in the endomembrane system and were associated with catalytic activity and anion binding (Figure 1C). KEGG analysis showed that these genes were mainly involved in metabolic pathways, steroid hormone biosynthesis and arachidonic acid metabolism (Figure 1D). The protein interaction network also showed complex correlations among these 58 genes (Figure 1E). These results suggest that lipid metabolism biological processes play an important role in high-risk NB.

### 2.2. Construction of a Prognostic Model for Lipid-Metabolism-Related Genes

**Figure 2:**
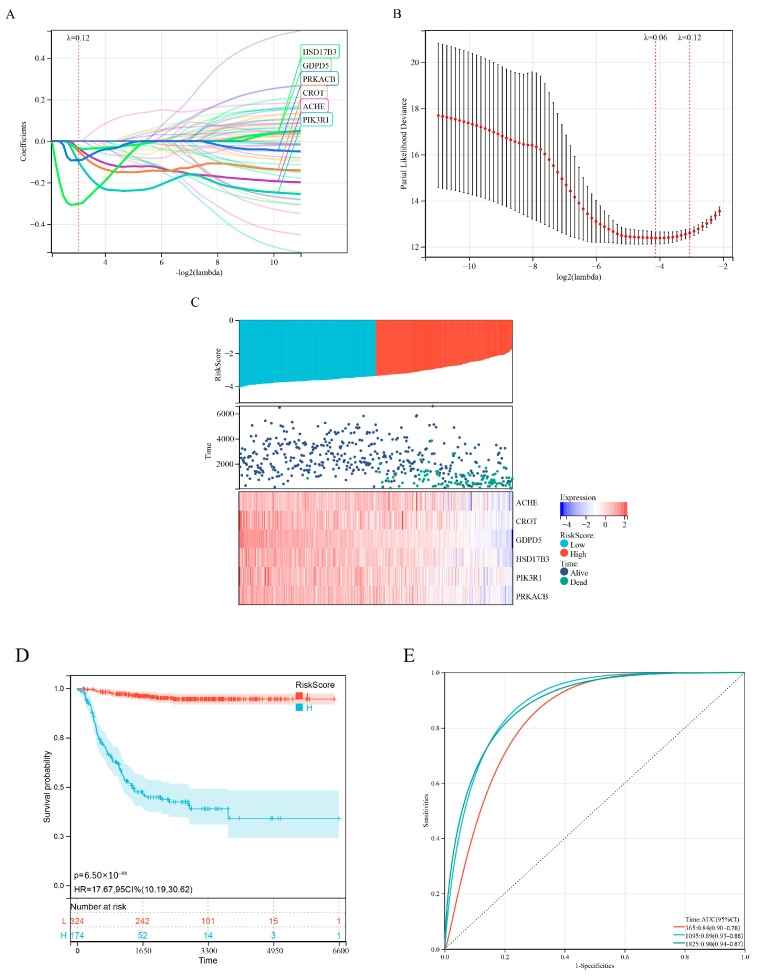
Development of a prognostic model for lipid metabolism genes. (**A**) Lasso coefficient profiles of 58 prognosis−related lipid metabolism genes from the Discovery (GSE49710 microarray) dataset. (**B**) Partial likelihood bias of the variable revealed by the Lasso regression model. The red dots represent the partial likelihood of deviated values, the grey line represents the standard error (SE) and the left and right vertical dashed lines represent the minimum standard and the best value for the 1−SE standard, respectively. (**C**) Expression heatmap of six signature genes, risk score distribution and survival status of patients. (**D**) Kaplan−Meier analysis of 6−gene risk scores. (**E**) ROC curve analysis of 6−gene risk score.

**Figure A1:**
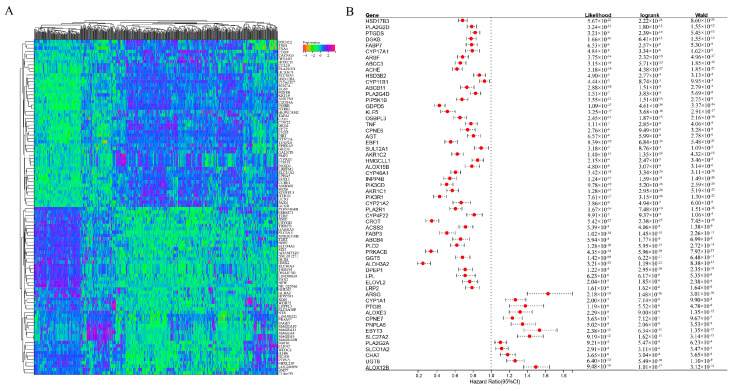
(**A**) Gene expression heatmap of Top–100 (up– and down–regulated) differentially expressed genes. (**B**) Prognostic survival analysis of 58 genes.

Difference analysis and survival analysis showed that these lipid-metabolism-related genes were significantly associated with NB risk (Figure A1A) and overall survival time (Figure A1B). Next, we performed LASSO Cox regression analysis to integrate survival time, survival status and gene expression data to obtain the optimal survival prediction model (Figure 2A–C). Finally, when the Lambda value is 0.120812060485213, the model formula constructed by six genes is obtained:

Risk Score = −a*ACHE−b*CROT−c*GDPD5−d*HSD17B3−e*PIK3R1−f*PRKACB.(1)

where:

a = 0.0345687415492563

b = 0.0489503629082964

c = 0.302176914910914

d = 0.0321116010448932

e = 0.0978743044989993

f = 0.0915003516148756.

**Figure A2:**
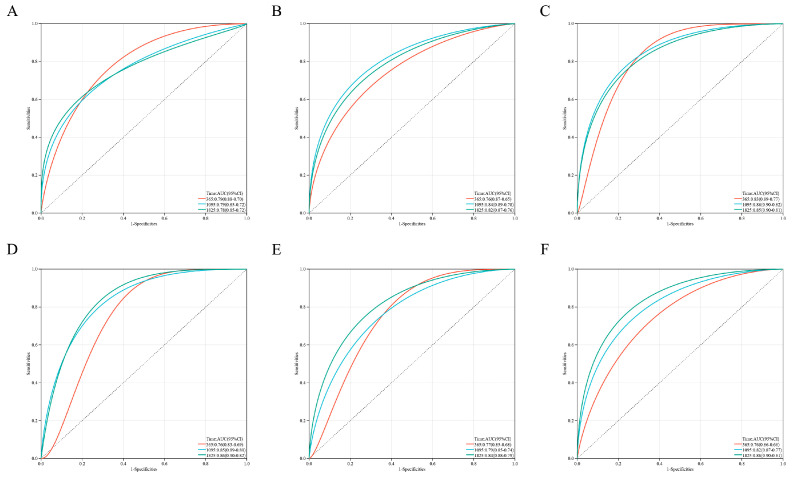
(**A**–**F**) is Kaplan–Meier analysis of ACHE, CROT, GDPD5, HSD17B3, PIK3R1, PRKACB in order.

Kaplan–Meier analysis showed that the risk score could effectively distinguish patients with better and worse prognosis (Figure 2D). ROC curve analysis (Figure 2E) showed that the area under the curve (AUC) values for the 1-, 3- and 5-year OS of the risk score were higher than those of individual genes (Figure A2), indicating that the prognostic power of risk score was better than that of individual genes alone.

### 2.3. Further Refinement of the Prognostic Model

**Figure 3:**
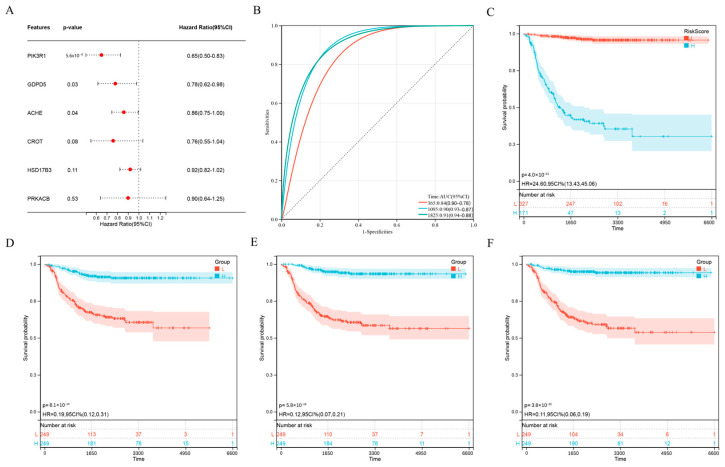
Further optimization of wind direction score. (**A**) Multivariate Cox regression analysis of 6 genes. (**B**) Kaplan–Meier analysis of novel 3-gene prognostic risk scores. (**C**) ROC curve analysis of novel 3-gene prognostic risk scores. (**D**–**F**) Kaplan–Meier analysis of ACHE (**D**), GDPD5 (**E**) and PIK3R1 (**F**).

To further optimize the prognostic model, we performed multivariate Cox analysis on the six genes (ACHE, CROT, GDPD5, HSD17B3, PIK3R1, PRKACB) in the risk score formula. The results showed that ACHE, GDPD5 and PIK3R1 were independent prognostic factors (Figure 3A). We then constructed a new risk score using these three genes:

Risk Score = ACHE*0.862854240578636 + GDPD5*0.777001638391889 + PIK3R1*0.646763659363925.(2)

where:

a = 0.862854240578636

b = 0.777001638391889

c = *0.646763659363925.

Interestingly, the ROC curve of the new scoring formula, composed of ACHE, GDPD5 and PIK3R1 (Figure 3B), was similar to the ROC curve composed of the previous six genes (Figure 2E) and better than the ROC curve of any single gene among them (Figure A2). In addition, the new scoring formula and ACHE, GDPD5 and PIK3R1 as independent prognostic factors can effectively distinguish populations with different prognosis (Figure 3C–F). This allows us to reduce the number of genes without affecting the AUC value, resulting in better clinical applicability.

Further, we investigated the optimal cutoff (Table 2) and median cutoff (Table 3) to evaluate the accuracy of risk score in distinguishing high-risk or non-high-risk (low and medium risk) children, respectively. The results showed that the optimal cut-off value grouping method had an accuracy of 85.23% and 93.48% in identifying children in the high-risk group and the non-high-risk group, respectively. The median stage grouping methods were 95.45% and 74.84%, respectively. The median grouping method was better at identifying high-risk children, but lower than the optimal cut-off grouping method at identifying non-high-risk children.

### 2.4. Independent Prognostic Factor GDPD5 Is Associated with Immune Infiltration

**Figure 4:**
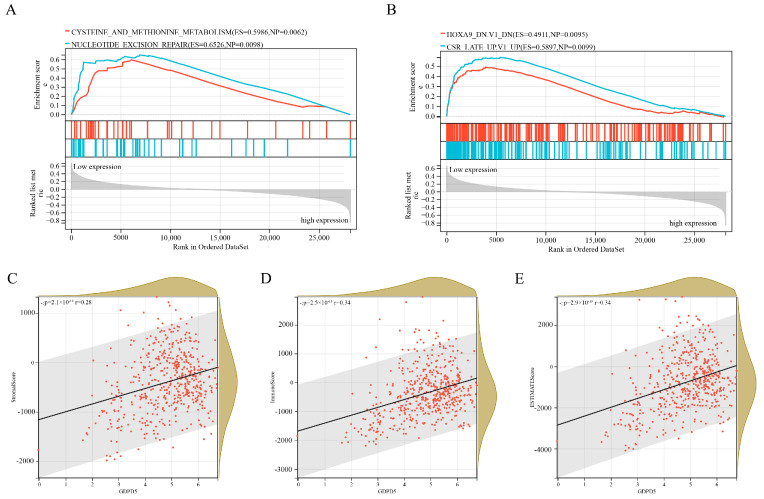
Gene Set Enrichment Analysis (GSEA) and immune infiltration analysis of GDPD5. (**A**) Two KEGG pathways enriched in samples with high GDPD5 expression. (**B**) Two KEGG oncology signatures enriched in samples with high GDPD5 expression. (**C**–**E**) The relationship between the expression of GDPD5 and stromal score (**C**), immune score (**D**) and estimate score (**E**). The horizontal axis represents the expression of GDPD5 and the vertical axis represents the score.

**Figure A3:**
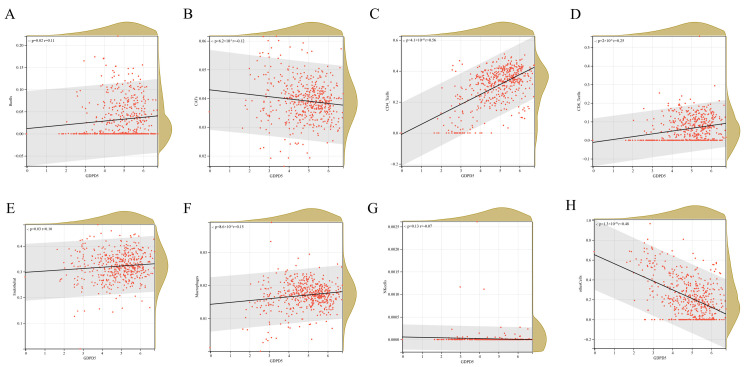
(**A**–**H**) is the relationship between the expression of GDPD5 and the infiltration of 8 kinds of immune cells. The horizontal axis is the expression of GDPD5 and the vertical axis is immune cells.

Among three important independent prognostic genes (ACHE, GDPD5, PIK3R1), GDPD5 caught our attention. GDPD5 was previously shown to promote neurogenesis and it was recently shown to promote NB cell differentiation in an autonomous manner [19]. We first examined oncological signatures and KEGG signaling pathways associated with GDPD5 expression. The results showed that samples with high GDPD5 expression were enriched in cysteine and methionine metabolism and nucleotide excision repair (Figure 4A), as well as two oncological features (Figure 4B). It is generally believed that the degree of immune cell infiltration is related to the occurrence, development, treatment and clinical prognosis of tumors [21,22]. The tumor microenvironment that constitutes various immune cell subsets influences the antitumor effect of immunotherapy [23]. Therefore, it is necessary to observe whether the expression of GDPD5 correlates with the immune infiltration of NB. Our results showed that GDPD5 was not only associated with stromal score (Figure 4C), immune score (Figure 4D) and estimate score (Figure 4E), but also with the majority of immune cell infiltration (Figure A3). These results suggest that GDPD5 is not only related to lipid metabolism in NB, but also to its immune status.

### 2.5. Hsa-miR-592 Is a Potential Target miRNA of GDPD5

**Figure 5:**
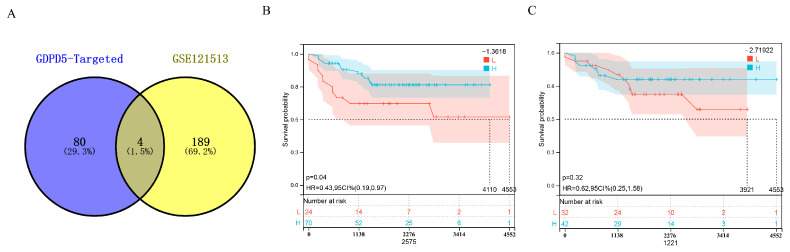
Identification of GDPD5-targeted miRNAs. (**A**) Venn diagram of differentially expressed miRNAs and GDPD5-targeted miRNAs. (**B**,**C**) Kaplan–Meier analysis of hsa-miR-592 (**B**) and hsa-miR-604 (**C**) in the GSE121513 dataset.

MicroRNAs (miRNAs) are small noncoding RNAs that broadly regulate gene expression in animals, plants and protozoa. miRNAs typically act post-transcriptionally by base-pairing to the 3′-untranslated region of mRNA to inhibit protein synthesis through a mechanism that is not fully understood [24]. First, we predicted GDPD5-targeted miRNAs in the miRSystem system (Table S2) and then identified 193 differentially expressed miRNAs (DEMs) in GSE121513 between patients in the high-risk group and non-high-risk group and obtained 2 miRNAs after taking the intersection upregulated DEM (hsa-miR-107, hsa-miR-592) and 2 downregulated (DEM) (hsa-miR-604, hsa-miR-636) (Figure 5A). Then, Kaplan–Meier analysis showed that hsa-miR-592 could effectively distinguish high-risk and low-risk groups (Figure 5B), but not hsa-miR-604 (Figure 5C). GDPD5 was down-regulated in high-risk patients, whereas miRNA-silencing mRNAs were generally expressed in the opposite manner. That is to say, the two down-regulated DEMs (hsa-miR-604 and hsa-miR-636) are not target miRNAs of GDPD5.

### 2.6. Overexpression of GDPD5 Affects Lipid Metabolism, Migration and Proliferation of SH-SY5Y Cell Line

**Figure 6:**
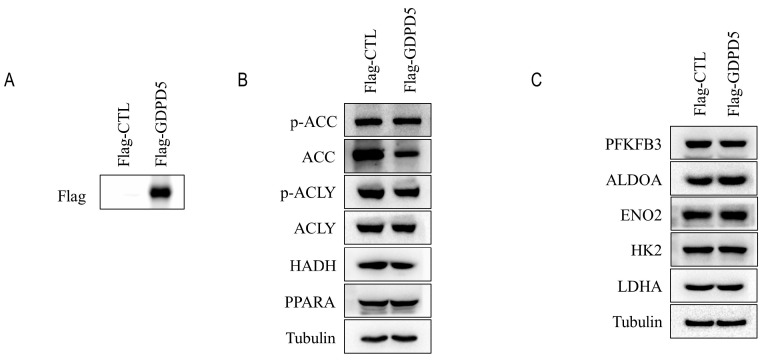
Immunoblot analysis. (**A**) Immunoblot analysis of transfected flag-GDPD5 or transfected flag-ctrl. (**B**) Western blot analysis of lipid-metabolism-related markers. (**C**) Western blot analysis of markers related to glucose metabolism. ACC (Acetyl-CoA Carboxylase Alpha), ACLY (ATP Citrate Lyase), HADH (Hydroxyacyl-CoA Dehydrogenase), Fructose-2,6-Biphosphatase 3 (PFKFB3), PPARA (Peroxisome Proliferator Activated Receptor Alpha), ALDOA (Aldolase, Fructose-Bisphosphate A), ENO2 (Enolase 2), HK2 (Hexokinase 2), LDHA (Lactate Dehydrogenase A).

**Figure 7:**
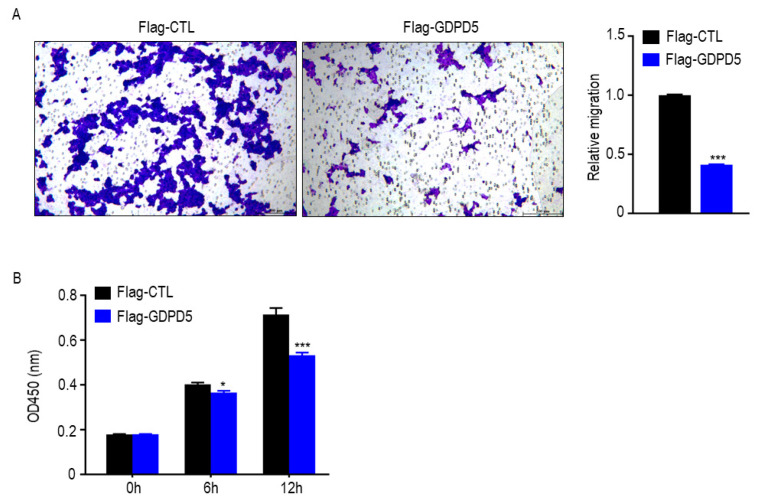
GDPD5 affects the migration and proliferation of human neuroblastoma cell line SH-SY5Y. (**A**,**B**) GDPD5 effects inhibit the migration (**A**,**B**) proliferation of SH-SY5Y. *: *p* < 0.05, ***: *p* < 0.001.

SH-SY5Y cells were established in 1970 and are often used as a cell model of neuroblastoma [25]. To further explore the biological function of GDPD5, we overexpressed GDPD5 in the human SH-SY5Y cell line (Figure 6A) and then examined the changes in its metabolism, as well as the migration and proliferation behavior of cancer cells. ACC, ACLY, HADH and PPARA are energy and lipid enzymes [26,27,28] and our Western blot results indicated that overexpression of GDPD5 resulted in a reduction in ACC (acetyl-coenzyme A carboxylase), a key enzyme in fat synthesis, but not the other (Figure 6B). Meanwhile, we detected changes in the glucose metabolism of SH-SY5Y by detecting PFKFB3, ALDOA, ENO2, HK2 and LDHA [29,30,31,32,33]. The results showed that overexpression of GDPD5 hardly affected the changes in SH-SY5Y glucose-metabolism-related markers (Figure 6C). Furthermore, overexpression of GDPD5 inhibited the migration and proliferation of SH-SY5Y cells (Figure 7). This suggests that GDPD5 may affect the lipid synthesis of the SH-SY5Y cell line by reducing ACC, thereby inhibiting the migration and proliferation of SH-SY5Y.

## 3. Discussion

According to the Children’s Oncology Group (COG) risk stratification, neuroblastoma (NB) can be divided into three grades: low, intermediate and high [34]. The five-year survival rate for low- and intermediate-risk patients is higher than 90%, while the long-term survival rate for high-risk patients is less than 50%, even after multiple approaches [35]. Therefore, it is necessary to explore new prognostic indicators to improve the prognosis of high-risk NB patients. The arrival of second-generation sequencing technology and the era of big data have provided new ideas for the prognosis evaluation of NB patients. Risk profiles related to MYCN [36,37], immunity [38] and glycosyltransferases are emerging [39]. Numerous studies have shown that lipid metabolism is related to cancer progression, metastasis and treatment and has the potential to become a new biomarker [40,41,42]. Risk scores of lipid-metabolism-related genes have been constructed to predict the survival rate of colon adenocarcinoma [43], lung adenocarcinoma [44], bladder cancer [5] and serous ovarian carcinomas [20]. However, the association between lipid-metabolism-related risk models and prognosis in NB patients remains unknown. To our knowledge, this study is the first to identify lipid-metabolism-related genes associated with NB prognosis using a bioinformatic approach. In this study, we identified 58 differentially expressed genes associated with risk stratification in NB patients in the GEO dataset (GSE49710). In addition, these genes were mainly enriched in steroid hormone biosynthesis and arachidonic acid metabolism. We identified a risk score, including ACHE, GDPD5 and PIK3R1, using LASSO and multivariate Cox regression analysis. Interestingly, GDPD5 was also included in the Nomogram constructed by Xia et al. [37]. However, the AUC values of our risk score ROC curves at 1, 3 and 5 years were 0.84, 0.90 and 0.91, respectively, which were higher than those of Xia et al. (0.754, 0.815, 0.795). This means that our three-gene risk score has better prognostic power.

GDPD5 maps to chromosome 11q13 and frequently shows loss of heterozygosity in high-risk NB [45], often associated with later stages and worse outcomes in NB [46]. GDPD5 induces NB cell differentiation and inhibits its motility through multiple mechanisms [19]. In this study, based on bioinformatics predictions, we identified hsa-miR-592 as a potential target miRNA of GDPD5. Our biological experiments showed that overexpression of GDPD5 would inhibit the expression of Acetyl-CoA Carboxylase Alpha (ACC, a key rate-limiting enzyme in lipid synthesis) in the human NB cell line SH-SY5Y, without affecting the expression of glucose-metabolism-related genes. In addition, overexpression of GDPD5 also inhibited the migration and proliferation of SH-SY5Y. Based on these results, GDPD5 will qualify as a potential tumor-suppressor gene. However, this study also has several shortcomings. First, the constructed risk scoring formula was not validated with a large sample. Second, the regulation of GDPD5 by hsa-miR-592 was not verified by biological experiments. Collectively, the findings of this study provide new insights into the occurrence and development of NB from the perspective of lipid metabolism. A prognostic model based on three lipid-metabolism-related genes can effectively predict the prognosis of NB patients. In addition, simple biological experiments suggest that GDPD5 affects the lipid metabolism, migration and proliferation of NB and is a potential biomarker for high-risk NB.

## 4. Materials and Methods

### 4.1. Data Extraction from Online Databases

The gene expression data and clinical information of GSE49710 and GSE121513 were downloaded from the GEO database (https://www.ncbi.nlm.nih.gov/geo/ (accessed on 23 May 2022)), based on the GPL16876 and GPL25696 platforms, respectively. GSE49710 has a total of 498 NB samples, including RNA-seq and clinical information (sex, age, International Neuroblastoma Staging System (INSS) stage, MYCN amplification status and overall survival) [47]. GSE121513 contains 117 samples with COG risk stratification and overall survival. Ensemble IDs were converted to corresponding gene symbols using the clusterProfiler R package. These gene expression matrices were then quantile normalized and log2 transformed for subsequent analysis.

### 4.2. Screening of Differentially Expressed miRNAs and Lipid Metabolism Genes

Differential expression analysis was performed on the gene expression matrix after GSE121513 and GSE49710 treatment to screen genes for differentially expressed miRNAs (DEMs) and lipid metabolism genes between high-risk and non-high-risk groups. The “limma” [48] R package was utilized and genes were identified as significant DEGs if |log2FC| value > 1 and false-discovery rate (FDR) < 0.05. Next, univariate Cox regression analysis was applied to identify DEGs associated with prognosis in NB patients.

### 4.3. Functional Enrichment Analysis and Protein–Protein Interaction Network Construction

This study used David (v.6.8, https://david.ncifcrf.gov/ (accessed on 23 May 2022)) to complete the enrichment analysis of DEGs, aiming to initially understand the biological functions of these genes. *p* < 0.05 and FDR < 25% were determined to be significant. The STRING (v 11.0, https://string-db.org (accessed on 23 May 2022)) database was used to construct protein–protein interaction (PPI) networks. Next, the networks were visualized using Cytoscape software (version 3.8.2).

### 4.4. Construction of a Prognostic Score for Lipid-metabolism-related Genes

LASSO is a shrinkage estimation method whose basic premise is to obtain a shrinkage subset by limiting the coefficients of some features to zero to minimize the residual sum of squares under the constraints. In this study, the LASSO Cox regression model in the “glmnet” R package was applied to identify DEGs associated with overall survival. Next, we obtain the beta coefficient risk score of each gene at the optimal λ value. The risk score is equal to the sum of the expression levels of each gene multiplied by the corresponding beta coefficients. Patients were divided into high-risk and low-risk groups according to the median cut-off value of risk score. The prognostic power of risk score was assessed by Kaplan–Meier analysis and ROC curve.

### 4.5. Gene Set Enrichment Analysis (GSEA) and miRNA Prediction

We performed GSEA on gene expression profiles based on high- and low-GDPD5 groups (https://www.gsea-msigdb.org/gsea (accessed on 23 May 2022)) [49]. First, we download a subset of c2.cp.kegg.v7.4.symbols.gmt to evaluate relevant pathways and molecular mechanisms. Then, we ranked by normalized enrichment score (NES) and visualize the results. *p* < 0.01, FDR < 0.25 was considered significant. MiRSystem (http://mirsystem.cgm.ntu.edu.tw/ (accessed on 23 May 2022)) was used to predict target miRNAs of GDPD5 [50].

### 4.6. Immune Infiltration and Immune Microenvironment Assessment

CIBERSORT (https://cibersortx.stanford.edu/ (accessed on 23 May 2022)) was used to assess the relationship between GDPD5 and the immune microenvironment [51]. ESTIMATE (https://sourceforge.net/projects/estimateproject/ (accessed on 23 May 2022)) was used to evaluate the relationship between GDPD5 and tumor immune infiltration [52]. EPIC (Immune Cell http://epic.gfellerlab.org/ (accessed on 23 May 2022)) was used to evaluate the relationship between GDPD5 and tumor immune cell content [53].

### 4.7. Plasmids Construction and Cell Culture Transfection

The GDPD5 (NM_001351167.2) coding sequences (CDS) were amplified with polymerase chain reaction (PCR) in vitro from the cDNA of HEK293T cells and were cloned into the CMV-flag vector by using restriction enzyme EcoRI and XhoI. The CMV-flag-GDPD5 plasmid was sequenced and aligned to confirm the DNA sequence was completely cloned (BGI, Chongqing, China).

The SH-SY5Y cell was grown in Dulbecco’s Modified Eagle Medium (DMEM) (Gibco, Carlsbad, CA, USA) supplemented with 10% fetal bovine serum (Gibco, Carlsbad, CA, USA) and 1% penicillin/streptomycin (PS) (Invitrogen, Grand Island, NY, USA). The cell was maintained at 37 °C in a 5% CO_2_ incubator. The Flag-CTL or Flag-GDPD5 plasmids were transfected into SH-SY5Y cells with Neofect™ DNA transfection reagent (Neofect biotech, Beijing, China), according to manufacturer’s protocol. Briefly, culture the SH-SY5Y cells with complete medium in 35 mm dish, dilute 2 μg DNA in Opti-MEM, add 2 μL Neofect™ gentle mixing, then incubate 25 min at RT and, finally, add transfection mix to plate and incubate at 37 °C in a 5% CO_2_.

### 4.8. Western Blot Analysis

The Flag-CTL and Flag-GDPD5 plasmids were transfected into SH-SY5Y cells with Neofect™ DNA transfection reagent. After cells were transfected for 48 h, the cells were lysed by 1% SDS lysing buffer containing Protease Inhibitor Cocktail and Phosphatase Inhibitor Cocktail (Apexbio, Houston, TX, USA) for Western blot analysis. The protein concentration was determined by a BCA protein assay reagent kit (Thermo Scientific, Waltham, MA, USA). All blots were, respectively, incubated with primary anti-bodies anti-flag (1:1000, ABclonal, Wuhan, China), anti-p-ACC, anti-ACC, Anti-p-ACLY, anti-ACLY, anti-PFKFB3, anti-HK2 and anti-LDHA (1:1000, Cell Signaling Technology, MA, USA), anti-ALDOA, anti-ENO2, anti-HADH and anti-PPARA (1:1000, proteintech, Wuhan, China), as well as anti-β-Tubulin (1:5000, TRANSGEN, Beijing, China). Bands were visualized with ECL Reagents (Smart-Lifesciences, Changzhou, China).

### 4.9. Transwell Assay

To confirm the effect of GDPD5 on SH-SY5Y cell migration, the Flag-CTL and Flag-GDPD5 plasmids were transfected into SH-SY5Y cells with Neofect™ DNA transfection reagent. After the cells were transfected for 48 h, a total of 1 × 10^4^ cells diluted in DMEM medium without FBS was plated into transwell chamber (Corning, NYS, USA), then the chamber was plated in 24-well plate supplemented with complete medium. After 24 h, the membrane was fixed with 4% paraformaldehyde for 30 min, washed with PBS for 5 min × 3, then the membrane was stained with 0.1% crystal violet (Sangon Biotech, Shanghai, China). Finally, the eight fields were captured randomly under a microscope (DMI8, Leica, Wetzlar, Germany). The quantitative analysis for migration was analyzed by Image J software (National Institutes of Health, Bethesda, MD, USA).

### 4.10. Cell Viability Assay

After cells were transfected for 48 h, the cell counting kit-8 (CCK-8) assay kits (MedChem Express, NJ, USA) were used for cell viability analyze. The Flag-CTL or Flag-GDPD5 cells were seeded in 96-well plates at a density of 2 × 10^4^ cells per well and cultured for 0 h, 6 h and 12 h. Then the DMEM medium (100 μL) and CCK8 solution (10 μL) were added to each well and incubated for 1 h. Finally, the absorbance of each well was measured at 450 nm using a microplate reader (Thermo Scientific, Waltham, MA, USA). A two-tailed paired or unpaired t-test statistical analysis was performed using GraphPad Prism 8 software (GraphPad, San Diego, CA, USA).

### 4.11. Statistical Analysis

R 4.0.2 was used for statistical analysis. The relationship between risk score or GDPD5 expression level and immune score, immune infiltration and immune cells was calculated using Spearman or Pearson correlation analysis. Kaplan–Meier analysis can compare survival status between groups. ROC curve analysis can test the predictive ability of the model. Unless otherwise stated, *p* < 0.05 was considered statistically significant.

## 5. Conclusions

Through various bioinformatic analyses of high-throughput sequencing datasets, we systematically assessed lipid-metabolism-related molecular features and prognostic value in NB and constructed a risk score composed of lipid-metabolism-related genes that independently predicted the prognosis of NB. It is biologically proved that GDPD5 is involved in lipid metabolism, migration and proliferation of NB, providing preliminary evidence for the complex biological function and immune regulation of GDPD5 involved in lipid metabolism in neuroblastoma. Our findings will help reveal the pathogenesis of NB and identify novel biomarkers and provide a basis for developing therapeutic strategies targeting lipid metabolism.

## Figures and Tables

**Table 1:** Clinical pathological parameters of NB patients in the training set.

Features	Number (%)
Sex	
Female	211 (42.4%)
Male	287 (57.6%)
Age	
≤18 months	300 (60.2%)
>18 months	198 (39.8%)
MYCN amplification	
No	401 (80.5%)
Unknown	5 (1.0%)
Yes	92 (18.5%)
INSS stage	
St1	121 (24.3%)
St2	78 (15.7%)
St3	63 (12.7%)
St4	183 (36.7%)
St4S	53 (10.6%)
Clinical risk	
High	176 (35.3%)
Low	322 (64.7%)
Class label	
Favorable	181 (36.3%)
Unfavorable	91 (18.3%)
Unknown	226 (45.4%)
Progression	
No	315 (63.3%)
Yes	183 (36.7%)
Death from disease	
No	393 (78.9%)
Yes	105 (21.1%)

**Table 2:** Accuracy evaluation of risk score formula grouping (optimal cutoff).

	Low Risk Score	High Risk Score	Accuracy
**COG (High)**	26	150	85.23%
**COG (Low and medium)**	301	21	93.48%
**Total**	327	171	498 (100%)

**Table 3:** Risk score formula grouping accuracy assessment (median).

	Low Risk Score	High Risk Score	Accuracy
**COG (High)**	8	168	95.45%
**COG (Low and medium)**	241	81	74.84%
**Total**	327	171	498 (100%)

## Data Availability

Data used in this study can be downloaded from Gene Expression Omnibus (GEO), NCBI’s publicly available genomics database (https://www.ncbi.nlm.nih.gov/geo/ (accessed on 23 May 2022)).

## Supplementary Materials

The supporting information can be downloaded at: https://www.mdpi.com/article/10.3390/ijms232213740/s1.

## Abbreviations

NB	Neuroblastoma
DEGs	Differentially Expressed Genes
PPI	Protein–protein interaction
FC	Fold Change
GEO	Gene Expression Omnibus
GO	Gene Ontology
KEGG	Kyoto Encyclopedia of Genes and Genomes
DAVID	Database for Annotation, Visualization and Integrated Discovery
STRING	Search Tool for The Retrieval of Interaction Genes
OS	Overall Survival
DFI	Disease free interval
DFS	Disease free survival
